# COVID-19 and Quitting Jobs

**DOI:** 10.3389/fpsyg.2022.916222

**Published:** 2022-06-16

**Authors:** Harun Demirkaya, Mustafa Aslan, Habibe Güngör, Vildan Durmaz, Didem Rodoplu Şahin

**Affiliations:** ^1^Uzunyol Vocational School, Kocaeli University, Kocaeli, Turkey; ^2^Faculty of Economics, Administration and Social Sciences, Istanbul Gelisim University, Istanbul, Turkey; ^3^Faculty of Aeronautics and Astronautics, Eskisehir Technical University, Eskişehir, Turkey; ^4^Faculty of Aeronautics and Astronautics, Kocaeli University, Kocaeli, Turkey

**Keywords:** COVID-19, quitting job, great resignation, depression, feeling of entrapment

## Abstract

Despite substantial studies on COVID-19 and the problems employees face, the association between COVID-19 and resigning jobs has not caught the interest of researchers. Millions have already resigned from their employment, and more are expected to resign. This study aims to investigate the relationship between the demographics of employees, the course of COVID-19, perceived effect of COVID-19 on life (PEoC), fear, entrapment feeling, depression, and quitting the job during the COVID-19 pandemic. A cross-sectional study was designed, and a convenient sampling method was adopted. Data were collected *via* an online questionnaire and analyzed by using SPSS version 26. Correlation and regression analyses were performed to reveal the relationship. Coefficients and significance values were used to interpret the results. Independent samples t-test and one-way ANOVA are used to determine the difference across the groups. The correlation between depression and work location is statistically significant. The PEoC increases fear, internal and external entrapment, and depression. Despite the statistically significant correlations between quitting jobs and the education level, internal and external entrapment, PEoC, fear, and depression for employees who have COVID-19 history, quitting the job was found to be affected only by COVID-19 history, internal entrapment feeling, and education level. This study has shown that quitting the job is associated with PEoC, depression, and internal and external entrapments. The correlation between quitting jobs and other conditions differs depending on the COVID-19 history of the employee. Furthermore, quitting the job is being affected by the coronavirus history, the internal entrapment, and education level.

## Background of the Study

A close friend of one of the authors of this article called in and asked his advice about quitting his job. Following a conversation about how he feels, the author started to question his friend’s feelings toward some aspects of work and private life. Afterward, the author interviewed over 20 people with COVID-19 history. It turns out that some of them had already quit their jobs, and some were in the process of seriously thinking about quitting. Only a few of the interviewees had never thought about quitting their jobs. Above all, the majority of the interviewees described their feelings with the words, “I feel like I’m being suffocated.” Observations led the authors to think that all interviewees showed feelings of entrapment and depression symptoms. Hence, the authors designed this study to investigate a possible correlation between COVID-19, feeling of entrapment, depression, and the quitting or intention to quit the job.

## Introduction

The COVID-19 pandemic emerged in Wuhan, China, in December 2019. Since its onset, it has caused a worldwide public health crisis and many lives (WHO, 2022) and made countless people go through traumatic experiences. The pandemic made almost everyone fear and suffer from trauma (Üngüren et al., 2022). Extended lockdowns, their negative impact on businesses, often ending up in bankruptcies (Boratyńska, 2021), shortage of food, and, most significantly, inability to access most basic healthcare services, fear of dying, or causing the death of a loved one, has increased the impact of this trauma.

People felt trapped in an unprecedented situation. No matter what precautions were taken, the spread of the coronavirus has left people with feelings of helplessness (Zhang and Ma, 2020) and being defeated (Chaturvedi et al., 2021). Moreover, the long lockdowns provided people with more time to spend on their electronic devices, enabling them to read more about the coronavirus, which increased worry and fear, leading to a downward spiral (Elhai et al., 2020), leaving no room for hope. As a result of the lockdowns and other measures implemented to halt the spread of the coronavirus, people have been restricted from engaging in physical and social activities, which might be regarded as key risk factors for both physical and physiological health (Soheili et al., 2020). To sum up, it has been an unpleasant and stressful scenario everyone tried to avoid but simply felt entrapped (Flett and Hewitt, 2020; Da-Silva-Lopes et al., 2021), which also provides an exact definition of the feeling of entrapment.

The feeling of entrapment is one of the psychological structures that play a major role in the occurrence of depression. Studies on the subject show that there are strong links between the feeling psychologically entrapped and depression (Taylor et al., 2011). The feeling of entrapment, which is a mood disorder, is a sense of being locked up while having a strong desire to escape from an unfavorable situation or a predicament. Being under stress and restricting behaviors are the factors triggering the feeling of entrapment. Meanwhile, the feeling of entrapment may also stem from subjective negative perceptions causing one to experience the sense of having no control over his conditions incessantly and inevitably (Taylor et al., 2011). Numerous variables can contribute to the sensation of entrapment, which plays a significant role in depression (Flett and Hewitt, 2020; Da-Silva-Lopes et al., 2021). There is a growing body of work studying people’s experiences of being defeated or stuck in various psychiatric conditions. Depression, anxiety issues, post-traumatic stress disorder (PTSD), and suicidality are the most commonly investigated disorders (Siddaway et al., 2015).

Many researchers have investigated the link between the entrapment feeling during COVID-19 and several other psychological concepts. For instance, in their research, Lee and Park (2021) found the following attributes of the entrapment feelings during the COVID-19: (1) being out of control, (2) having no escape, (3) being trapped, (4) being robbed, and (5) hopelessness. The reasons for these feelings were identified as: (1) the COVID-19 pandemic itself, (2) lockdowns put in place by states, (3) restrictions, (4) uncertainty about the future, (5) economic hardships, and (6) a poor ability to deal with the situation. Meanwhile, the outcomes of the entrapment were found as follows: (1) increased number of suicides, (2) deteriorating mental health, and (3) poorer well-being.

Despite the extensive studies and the problems that employees face, the relationship between the COVID-19 and quitting jobs has not attracted researchers. Millions of people have already resigned from their jobs because of the consequences of the pandemic. Over 24 million Americans resigned between April and September 2021 (Bloomberg Businessweek, 2021), and about 4.5 million in November 2021 (Davidson, 2021), and expected many more to resign (Bloomberg Businessweek, 2021; Davidson, 2021). A 21% of doctors consider leaving National Health Service (Sheather and Slattery, 2021) in the United Kingdom. The severity of the problem may be better understood if we consider the rest of the world. This situation is called Great Resignation (Bloomberg Businessweek, 2021; Davidson, 2021; Sheather and Slattery, 2021).

## The Purpose of the Study

Although many organizations around the globe suffer from the great resignation (Bloomberg Businessweek, 2021; BMA, 2021; Chugh, 2021; Davidson, 2021; Sheather and Slattery, 2021; Sull et al., 2022), there are scarce studies on the issue. The causes of the great resignation and the relationships with other individual and organizational conditions are yet to be investigated. As mentioned in the literature, resignations may be due to burnout syndrome (BMA, 2021; Chugh, 2021; Sheather and Slattery, 2021) or toxic organizational culture (Sull et al., 2022). However, long lockdowns, restrictions, and uncertainty, which cause feelings of being trapped and hopelessness associated with mental health problems (Lee and Park, 2021), may also be associated with resignations.

Therefore, our research questions are as “*does the COVID-19 pandemic have an association with fear, PEoC, depression, entrapment, and ultimately quitting jobs? If it does, then is there any relation with the demographics of the employees?*”

Hence, this study aims to fill the gap mentioned above by investigating the relationship between the demographics of employees, courses of COVID-19, PEoC, fear, entrapment feeling, depression, and quitting the job during the COVID-19 pandemic.

## Materials and Methods

In this study, 24 people with a history of COVID-19 were interviewed. The interviews were carried out to understand the feelings of people with COVID-19 history toward their jobs. Some of those who were interviewed had already quit their jobs, and some were in the process of seriously thinking about quitting. Only a few of them had never thought of quitting, and the majority of them described their feelings as: “I feel like I’m being suffocated,” and more than half of them reported that their job has “no meaning” for them anymore. The interviewees showed the feeling of entrapment and depression symptoms. Hence, the researchers designed this study according to the findings of those interviews to find the relationship between entrapment feeling, depression, quitting the job, and other contextual conditions, such as the course and the effect of the disease, fear experienced, and the work location.

### Data Collection

This cross-sectional online survey was conducted between September and October 2021 in Istanbul, the Republic of Turkey. The survey was designed to obtain the level of fear employees have about the consequences of coronavirus, perceived effect of coronavirus on their life (PEoC), work location, entrapment, and depression levels of the employees with and without coronavirus history.

We reached the participants through social media like LinkedIn and WhatsApp groups and provided the questionary links to those who expressed interest in taking the survey (a total of two links: one for those with coronavirus history and one for those with no history). A total of 321 people requested the links, but only 243 of them filled out the survey (75.7% response rate). The criteria for inclusion are to be an employee or used to be an employee and 18 years old or older.

The data collection process took less than 10 min for each participant.

### Measures

The questionary consisted of four parts. The questions in the first part of the questionary were about the demographics of the participants, such as age, gender, and workplace during the pandemic (e.g., working from home, both from home & workplace, and only from the workplace). In the second part, we asked the participants if they had lost any relatives or close friends due to coronavirus. We also asked if they had quit or are thinking to quit their jobs and what they fear most if they get infected with the coronavirus. In the third part, we used two different scales: (1) the 21-item Beck Depression Inventory-II (Beck et al., 1996), (2) the entrapment scale developed by Gilbert and Allan (1998). The scores of Beck Depression Inventory-II were interpreted as suggested by Smarr and Keefer (2011): minimal range = 0–13, mild depression = 14–19, moderate depression = 20–28, and severe depression = 29–63.

Some items of the Beck Depression Inventory-II are as:

- 0 I do not feel sad.
  1 I feel sad.
  2 I am sad all the time and I cannot snap out of it.
  3 I am so sad and unhappy that I cannot stand it.
- 0 I am not particularly discouraged about the future.
  1 I feel discouraged about the future.
  2 I feel I have nothing to look forward to.
  3 I feel the future is hopeless and that things cannot improve.
  Some of the items from the entrapment scale are as:

- Internal Entrapment
  1- I want to get away from myself.
  2- I feel powerless to change myself
- External Entrapment
  2-I have a strong desire to escape from things in my life.
  3-I am in a relationship I cannot get out of.
  Participants with a COVID-19 history were also asked about the course of the COVID-19.

#### Fear

We asked respondents to assess the following five items on a scale of 1 to 5 to measure the reason for their fear of contracting coronavirus (1 is the lowest; 5 is the highest level of fear). The five items were related with:

- Afraid of being infected with the coronavirus.
- Afraid of infecting his/her family members or loved ones.
- Afraid of infecting people other than his/her family members and loved ones.
- Afraid of losing someone because of infecting him/her with the disease.
- Afraid of dying.

An exploratory factor analysis (EFA) was performed using the principal components extraction method. Bartlett’s test of sphericity was found to be significant [*χ*^2^ (10) = 847.946, *p <* 0.001]. The Kaiser-Meyer-Olkin measure of sampling adequacy was high (KMO = 0.819). Thus, proceeding with the analysis was considered to be acceptable. A varimax rotation method was performed, and only one factor with the Eigenvalue greater than one was extruded. All factor loadings were greater than 0.85 except the first item, which had a factor loading of 0.453. The extracted factor accounted for 68.759% of the variance in the data.

#### Intention to Quit

The question to assess the intention to quit was the following: “Have you ever thought of quitting your job because of COVID-19?” The options presented to the participants were as: (1) I have never thought of quitting my job because of COVID-19, (2) I have thought of quitting my job because of COVID-19 but not very often, (3) I have thought of quitting my job because of COVID-19 very often, (4) I am seriously thinking of quitting my job because of COVID-19, and (5) I have already quit my job because of COVID-19.

The question “How did the COVID-19 pandemic affect the quality of your life?” was asked to assess the PEoC. They were presented with the following options: 1: not affected at all; 2: minimal adverse effect; 3: moderate adverse effect; and 4: very high adverse effect.

Participants marked 0 if they had not lost any relatives or close friends, 1 for one relative or close friends, and 2 for more than one relative and close friends.

For the course of the COVID-19, participants were given six options: (1) no symptoms, (2) mild, (3) moderate, (4) severe but stayed at home, (5) severe and hospitalized, and (6) severe and needed intensive care.

### Data Analysis

The data were analyzed by using SPSS version 26. We used descriptive statistics to report the frequencies and performed regression and correlation analysis to reveal the relationship between variables. Coefficients and significance levels were used to interpret the results. The statistical significance of the difference across the groups was also analyzed using independent samples *t*-test for constructs consisting of two groups and one-way ANOVA for more than two groups.

Analysis was conducted for total samples and participants with COVID-19 history, respectively.

## Results

Out of a total of 243 surveys, 237 were included in the statistical analyses. Six surveys—two of whom belonged to underaged participants and four of them housewives—were excluded because they did not meet the criteria. The average age of respondents was 40.17. Table 1 shows the demographics of the participants.

**Table 1:** Demographics of the participants.

Demographics	Options	Coding	Frequency	Percent
COVID History	No	0	102	43.0
	Yes	1	135	57.0
Age	25 and below	1	22	9.3
	26–30	2	37	15.6
	31–35	3	28	11.8
	36–40	4	39	16.5
	41–45	5	26	11.0
	46–50	6	39	16.5
	51–55	7	25	10.5
	56 and above	8	21	8.9
Gender	Male	1	101	42.6
	Female	2	136	57.4
Decease	None	0	125	52.7
	One	1	53	22.4
	Two or More	2	59	24.9
Education level	High-School and Below	1	34	14.3
	University	2	120	50.6
	MSc Degree	3	51	21.5
	Ph.D.	4	32	13.5
PEoC	Not effected	0	124	52.3
	Minimal Adverse Effect	1	35	14.8
	Moderate Adverse effect	2	51	21.5
	Very High Adverse effect	3	27	11.4
Work location	Home only	1	45	19.0
	Both home & workplace	2	88	37.1
	workplace only	3	104	43.9
	Total		237	100.0

**Figure 1:**
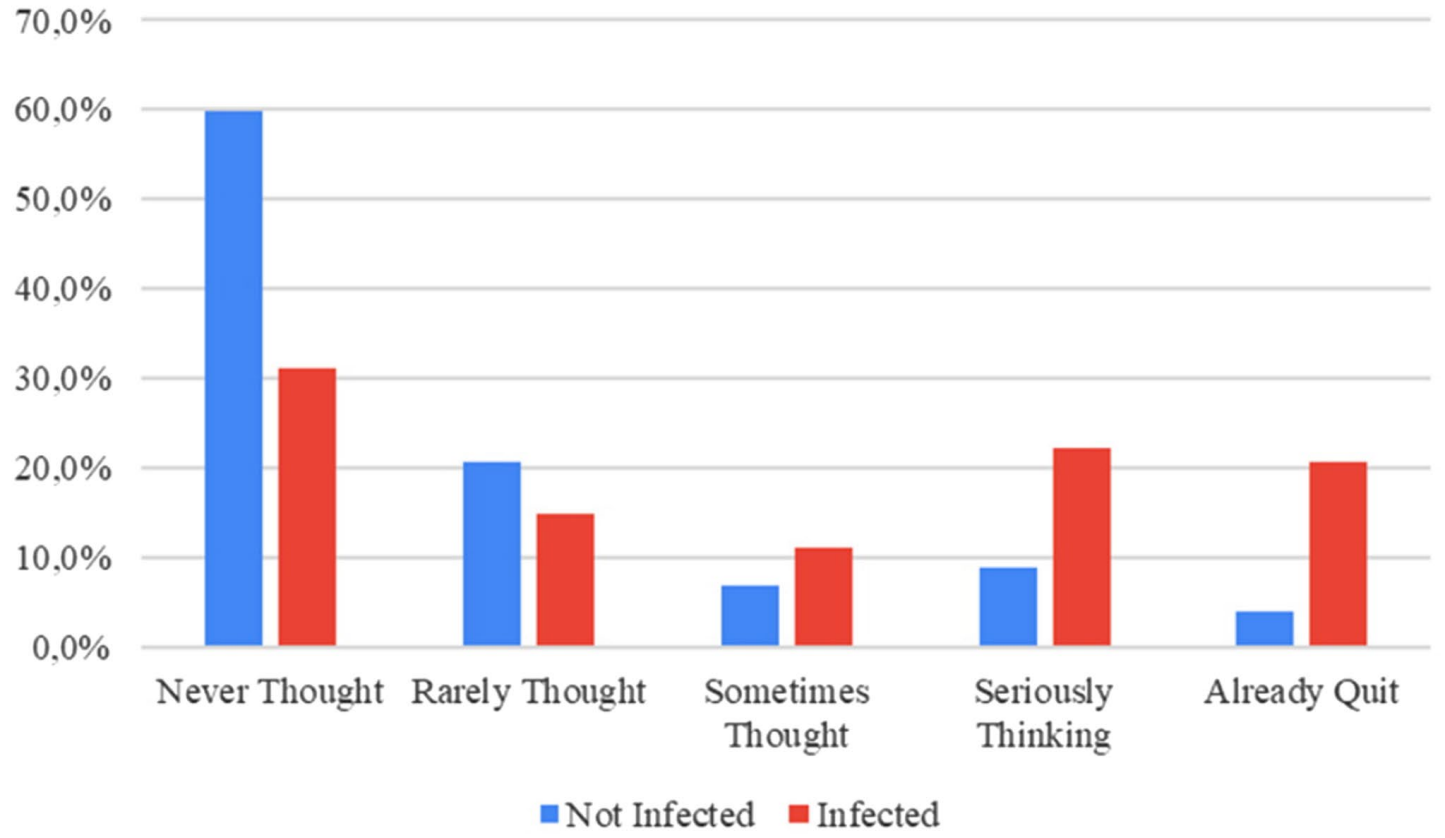
Quitting Jobs.

Out of 135 infected people: 42 people (%31.1) had never thought of quitting their jobs, while 28 people (%20.7) already had quit. Out of 102 uninfected people: 61 people (%59.8) had never thought of quitting their jobs, while 4 (%3.9) had already quit. All the figures related to quitting jobs are given in Table 2; Figure 1.

**Table 2:** Quitting jobs.

	Uninfected		Infected	
	*f*	%	*f*	%
Never thought	61	59.8%	42	31.1%
Rarely thought	21	20.6%	20	14.8%
Sometimes Thought	7	6.9%	15	11.1%
Seriously Thinking	9	8.8%	30	22.2%
Already quit	4	3.9%	28	20.7%

Around 21% of the respondents with COVID-19 history had already quit their jobs. This figure is almost six times higher than uninfected respondents. Moreover, the percentage of people seriously thinking of quitting is nearly three times more than those uninfected. Around 43% of the people with COVID-19 history have either already quit or are seriously thinking of quitting their jobs.

During the interview, we inquired into the reason for quitting the job. The majority of the answers were almost the same: “I feel like I’m being suffocated.” A participant said working was no longer bearable for him. Another one, who lost his uncle because of COVID-19, said she transmitted the disease to her uncle after contracting it from her workplace; she was blaming herself for his death. A person who was seriously thinking of quitting said he is dragging his feet when he leaves home for work.

None of the respondents had to go through intensive care due to COVID-19; 14 of them (10.4%) were hospitalized, the conditions of 49 of them (36.3%) were severe but received treatment at home; and 72 of them (53.3%) suffered either from moderate or mild symptoms or had no symptoms at all. The figures related to the course of COVID-19 are given in Table 3.

**Table 3:** The course of the COVID-19.

	*f*	%
No symptoms	15	11.1%
Mild	31	23.0%
Moderate	26	19.3%
Severe (at home)	49	36.3%
Severe (hospitalized)	14	10.4%
Intensive care	0	0.0%

The mean value of fear was measured as 4.0153 out of 5 point scale. The mean and standard deviations of each item in the fear of coronavirus scale are given in Table 4.

**Table 4:** Fear of respondents.

Item	*N*	Min.	Max.	*M*	SD
Afraid of dying.	237	1.00	5.00	2.7089	1.53064
Afraid of infecting people other than his/her family members and loved ones.	237	1.00	5.00	4.2785	1.11921
Afraid of being infected with the coronavirus.	237	1.00	5.00	4.2827	1.19685
Afraid of infecting his/her family members or loved ones.	237	1.00	5.00	4.4008	1.07931
Afraid of losing someone because of infecting him/her with the disease.	236	1.00	5.00	4.4153	1.08629
**FEAR**	**236**	**1.00**	**5.00**	**4.0153**	**0.96055**
Valid *N* (listwise)	236				

The bold line is the total score of the fear scale while the lines above that bold line are the values for each item in the scale.

As per descriptive statistics given in Table 4, the highest fear that respondents had was causing the death of someone because of infecting him/her (*M* = 4.4153), while the lowest was dying (*M* = 2.7089).

The results suggest that people do not consider themselves to be in the risk group or believe they would not die of coronavirus. They were simply afraid of causing the death of someone, be it a family member or not. People with a history of coronavirus, on the other hand, had a higher average in both *Afraid of infecting his/her family members or loved ones* (Δ*M* = 0.30; *p* = 0.044) and *Afraid of losing someone because of infecting him/her* (Δ*M* = 0.51; *p* = 0.001). Nonetheless, the mean difference of fear as a variable was not statistically significant (*p* = 0.112) among the coronavirus history groups. The mean differences between work-location groups were also not statistically significant.

Depression scores of the participants were also measured and reported in Table 5.

**Table 5:** Depression scores of the respondents.

	Infected		Uninfected		Overall	
	*f*	%	*f*	%	*f*	%
Minimal range (0–13)	95	70	72	71	163	69
Mild depression (14–19)	16	12	16	16	33	14
Moderate depression (20–28)	16	12	9	9	27	11
Severe depression (29–63)	8	6	5	5	14	6

As shown in Table 5, only 69% of participants were not in depression. Only 14 participants (6%) suffered from severe depression, while 27 (11%) of them suffered from moderate and 33 (14%) of them from mild depression. Nevertheless, neither the mean difference across age groups [*F*(8,126) = 1.496; *p* = 0.165] nor between infected and uninfected participants (*p* = 0.527) were statistically significant.

Correlation results with Pearson correlation coefficients and significance levels are given in Table 6. As per the results given in Table 6, depression and age are negatively correlated (*r* = −0.182; *p <* 0.01), which means the depression score elder people have lower depression scores.

**Table 6:** Correlations.

S. No.		1	2	3	4	5	6	7	8	9	10	11	12
1.	COVID History	1											
2.	Age	−0.112	1										
3.	Gender	0.026	−0.322^**^	1									
4.	Education	0.085	0.100	0.015	1								
5.	Work location	0.152^*^	0.006	−0.066	−0.083	1							
6.	Loss of life	−0.065	0.242^**^	−0.124	−0.088	0.097	1						
7.	PEoC	0.515^**^	0.017	0.117	−0.007	0.117	0.142^*^	1					
8.	Fear	0.101	−0.013	0.001	0.081	−0.043	0.109	0.245^**^	1				
9.	Internal Entrapment	−0.068	−0.100	0.184^**^	−0.044	−0.008	0.152^*^	0.372^**^	0.320^**^	1			
10.	External Entrapment	0.054	−0.102	0.165^*^	0.012	0.105	0.133^*^	0.389^**^	0.295^**^	0.723^**^	1		
11.	Depression	−0.065	−0.182^**^	0.215^**^	−0.126	−0.091	0.080	0.245^**^	0.233^**^	0.711^**^	0.525^**^	1	
12.	Quitting Job	0.364^**^	0.015	0.051	0.177^**^	−0.027	0.027	0.342^**^	0.219^**^	0.284^**^	0.307^**^	0.209^**^	1

Numbers are Pearson correlations coefficients; *N* = 237.

* *p* < 0.05;

** *p* < 0.01.

Internal (*r* = 0.184; *p <* 0.01) and external (*r* = 0.165; *p <* 0.05) entrapment feelings and depression levels (*r* = 0.215; *p <* 0.01) of females are higher than males.

As per the results given in Table 6, there is a correlation between work location and the transmission of coronavirus. Only 14.8% of the infected employees were working from home. A 35.6% of them worked from both home and workplace, while 49.6% of them were working only from the workplace. However, no correlation was found between work location and quitting jobs (*r* = −0.027; *p* > 0.05). As far as concerning the comparison of employees with (*M* = 2.8741; SD = 1.15335) and without (*M* = 1.7647; SD = 1.58081) coronavirus history, the mean differences of Quitting Jobs is found to be statistically significant (Δ*M* = −1,10,937; ΔSD = 0.18538; *p* = 0.000), which means that employees with coronavirus history had quit or intention to quit the job compared those without coronavirus history.

The PEoC was found to be correlated with COVID-19 history (*r* = 0.515; *p <* 0.01). The mean difference of the PEoC of employees with COVID-19 history (*M* = 1.4074) and other employees (*M* = 0.2745) is statistically significant (a higher value means a worse effect). The employees with COVID-19 history are the ones who have the worst PEoC. The PEoC is also positively associated with fear (*r* = 0.245; *p <* 0.01), internal (*r* = 0.372; *p <* 0.01) and external (*r* = 0.389; *p <* 0.01) entrapment, depression (*r* = 0.245; *p <* 0.01), and quitting job (*r* = 0.342; *p <* 0.01). The mean difference in quitting jobs across PEoC groups is also statistically significant (*F*(3,233) = 10.961; *p* = 0.000). The means of PEoC of moderate and highly affected groups are significantly higher than the no-effect and mildly affected groups.

Furthermore, the mean differences of those two groups (e.g., moderately and highly affected groups) are statistically higher than the rest for fear [*F*(3,332) = 5.662; *p* = 0.001], internal [*F*(3,333) = 15.715; *p* = 0.000] and external [*F*(3,333) = 14.337; *p* = 0.000] entrapment, and depression [*F*(3,333) = 7.529; *p* = 0.000].

The correlation between education level and quitting jobs is statistically significant [*F*(3,233) = 2.975;*p* = 0.032]. The mean difference in quitting jobs of employees with Ph.D. degrees (*M* = 2.9688) and MSc degrees (*M* = 2.6471) is significantly higher than university (*M* = 2.1917) and high-school graduates (*M* = 2.2059).

**Figure 2:**
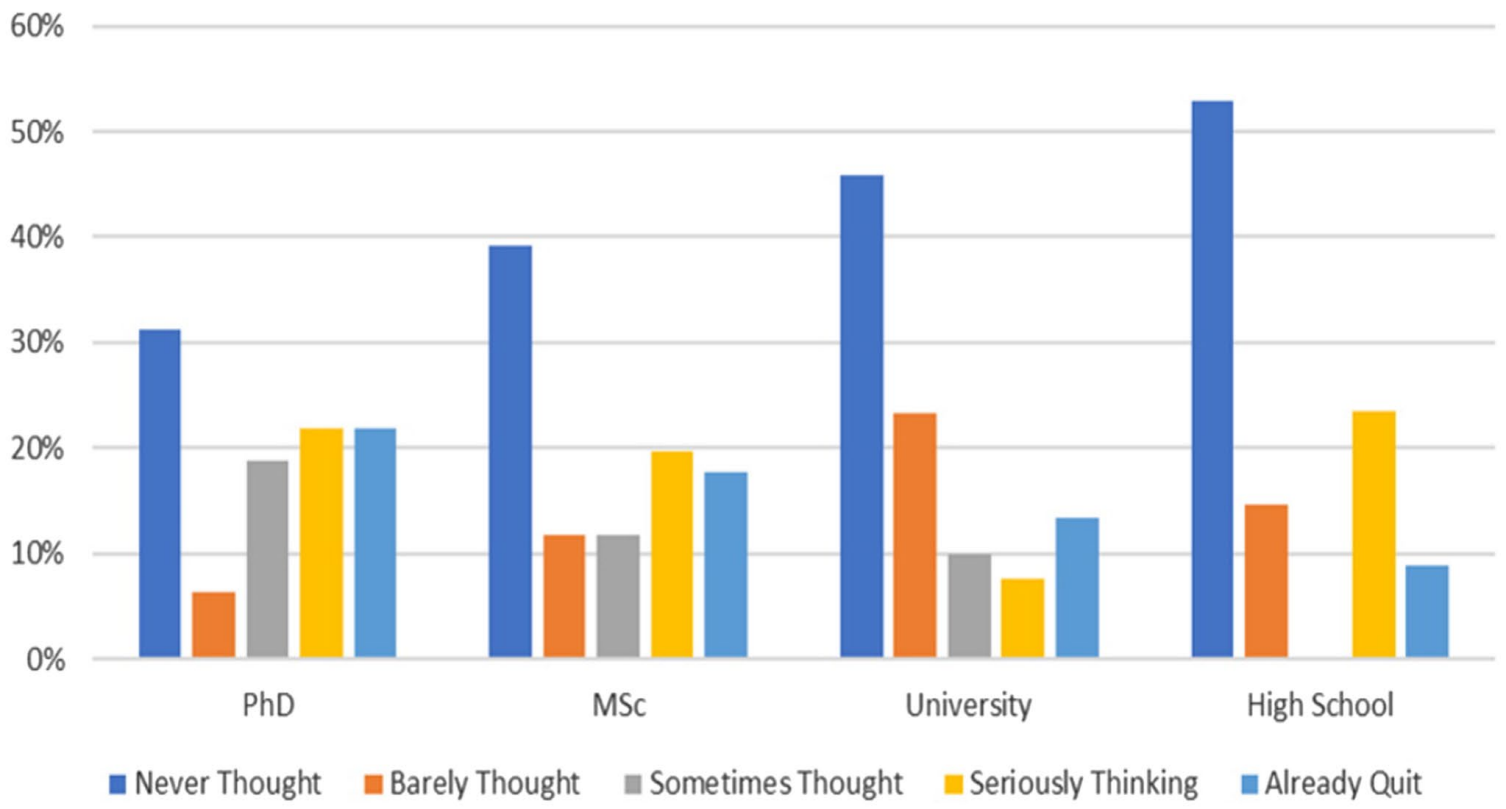
Quitting jobs per education level.

Figure 2 illustrates employees’ attitudes toward their jobs; 22% of Ph.D., 18% of MSc, 13% of university, and 9% of high-school graduates had already quit.

Fear is found to be associated with internal (*r* = 0.320; *p <* 0.01) and external (*r* = 0.295; *p <* 0.01) entrapment, and depression (*r* = 0.233; *p <* 0.01). Quitting job was correlated with fear (*r* = 0.219; *p <* 0.01) as well. Quitting job was found to be correlated with internal (*r* = 0.284; *p <* 0.01) and external (*r* = 0.307; *p <* 0.01) entrapment and depression (*r* = 0.209; *p <* 0.01) as well. The highest correlation to quitting job was measured with COVID-19 history (*r* = 0.364; *p <* 0.01), PEoC (*r* = 0.342; *p <* 0.01), and then external entrapment (*r* = 0.307; *p <* 0.01).

### Comparing the Infected and Uninfected Employees

The correlation analysis results for employees infected with coronavirus are reported in Table 7. The results of those uninfected are in Table 8.

**Table 7:** Correlation table for employees with COVID-19 history.

S. No.		1	2	3	4	5	6	7	8	9	10	11	12
1.	Age	1											
2.	Gender	−0.326^**^	1										
3.	Education	0.170^*^	−0.052	1									
4.	Work Location	−0.061	0.031	−0.134	1								
5.	Loss of death	0.244^**^	−0.134	−0.089	0.167	1							
6.	PEoC	0.144	0.204^*^	0.012	0.089	0.213^*^	1						
7.	Fear	0.083	0.070	0.130	−0.002	0.245^**^	0.319^**^	1					
8.	Internal Entrapment	−0.057	0.229^**^	0.008	0.055	0.131	0.597^**^	0.249^**^	1				
9.	External Entrapment	−0.091	0.211^*^	0.015	0.086	0.137	0.535^**^	0.207^*^	0.700^**^	1			
10.	Depression	−0.065	0.256^**^	−0.120	0.036	0.126	0.558^**^	0.213^*^	0.732^**^	0.518^**^	1		
11.	Quitting job	0.138	0.009	0.248^**^	−0.098	0.037	0.221^*^	0.191^*^	0.269^**^	0.234^**^	0.185^*^	1	
12.	Course of disease	0.165	0.163	0.066	−0.042	0.222^**^	0.451^**^	0.335^**^	0.315^**^	0.236^**^	0.239^**^	0.051	1

Numbers are Pearson correlations coefficients; *N* = 237.

* *p* < 0.05;

** *p* < 0.01.

**Table 8:** Correlation table for employees with NO COVID-19 history.

S. No.		1	2	3	4	5	6	7	8	9	10	11
1.	Age	1										
2.	Gender	−0.321^**^	1									
3.	Education	0.053	0.109	1								
4.	Work location	0.148	−0.197^*^	−0.048	1							
5.	Loss of life	0.226^*^	−0.108	−0.076	0.038	1						
6.	PEoC	0.020	−0.016	−0.197^*^	−0.020	0.199^*^	1					
7.	Fear	−0.038	−0.065	−0.192	−0.019	−0.011	0.009	1				
8.	Internal Entrapment	−0.156	−0.029	0.031	−0.093	−0.060	0.025	0.137	1			
9.	External Entrapment	−0.076	−0.053	0.023	−0.101	0.033	0.072	0.088	0.889^**^	1		
10.	Depression	−0.352^**^	0.162	−0.141	−0.205^*^	0.021	0.000	0.114	0.273^**^	0.156	1	
11.	Quitting job	−0.075	0.050	0.086	−0.021	0.116	0.252^*^	0.167	0.297^**^	0.223^*^	0.430^**^	1

Numbers are Pearson Correlations Coefficients; *N* = 237.

* *p* < 0.05;

** *p* < 0.01.

As per the results reported in Table 7, there is no correlation between work location and quitting jobs even for infected employees (*r* = −0.098; *p* = 0.258 > 0.05). The correlation between depression and age lost its significance (*r* = −0.065; *p* = 0.454 > 0.05) which was statistically significant (*r* = −0.182; *p <* 0.01) for the whole sample as per Table 6 and significant for uninfected employees as per Table 8. These results suggest that depression levels of uninfected younger employees are higher than others. In fact, the mean depression level of the uninfected employees below the age of 25 falls into the mild depression level range (*M* = 15.222). The means of the rest of the age groups are below 10; even the mean depression level of age group *56 and over* is below 5. The mean depression level of the infected employees changes between 13 (for the age group of 51–55) and 9 (for the age group of 56 and over).

The correlation between the gender and PEoC of the infected employees is significant (*r* = 0.204; *p <* 0.05), which was insignificant (*r* = 0.117; *p* > 0.05) for the whole sample as per Table 6 and still insignificant for the uninfected employees (*r* = −0.016; *p* > 0.05) as per Table 8. This result suggests that, when infected with COVID-19, the PEoC of women is affected worse than men.

The Pearson correlation coefficient and significance levels show that there are correlations between gender and internal entrapment (*r* = 0.229; *p <* 0.01), external entrapment (*r* = 0.211; *p <* 0.01), depression (*r* = 0.256; *p <* 0.01) for employees with coronavirus history (Table 7), and no correlation for employees uninfected with coronavirus (Table 8).

As per Tables 7, 8, the PEoC is negatively correlated with education level for those employees who are not infected, while there is no correlation for employees with COVID-19 history. There are no statistically significant differences across the education groups. This result suggests that the employees with higher education levels may feel the restrictions the disease puts on their lives more than others.

The statistically significant correlation that exists between education level and quitting jobs (*r* = 0.248; *p <* 0.01) for employees with coronavirus history could not be observed for uninfected employees (*r* = 0.086; *p* > 0.05). No research in the literature supports this finding. This situation may be due to the employees’ financial status and savings. The level of income is closely related to the education level. The employees with a higher level of education may have savings upon which they can rely until the end of the pandemic, or it is taken under control, enabling them to consider quitting their jobs.

The correlation between depression and work location is statistically significant (*r* = −0.205; *p <* 0.05) for not infected employees and not significant (*r* = 0.036; *p* > 0.05) for employees with coronavirus history. The mean difference across groups is significant [*F*(2,99) = 3.999; *p* = 0.021], and the mean depression level of the employees working only from home (*M* = 14.6400) is significantly higher than that of those working from both home and workplace (*M* = 8.6250) and only from the workplace (*M* = 9.3243).

The course of the disease is highly correlated with the PEoC (*r* = 0.451; *p <* 0.01), depression (*r* = 0.239; *p <* 0.01), fear (*r* = 0.335; *p <* 0.01), and internal (*r* = 0.315; *p <* 0.01) and external (*r* = 0.236; *p <* 0.01) entrapment. However, it has no correlation with quitting jobs (*r* = 0.051; *p* > 0.05).

The fear (r = 0.319; *p <* 0.01), internal (*r* = 0.597; *p <* 0.01) and external (*r* = 0.535; *p <* 0.01) entrapment, and depression score (*r* = 0.558; *p <* 0.01) are statistically significant for employees with coronavirus history and insignificant for uninfected employees.

### Regression Analysis

The above-mentioned independent variables were also entered into a regression analysis in SPSS to determine the predictors of quitting jobs (Table 9).

**Table 9:** Predictors of quitting jobs.

Independent variables	Standardized coefficient (*t*-value)
Age	0.094 (1.445)
Gender	0.007 (0.117)
Education	0.147 (2.473)^*^
Work location	−0.073 (−1.236)
Course	−0.188 (−1.512)
PEoC	0.067 (0.833)
Decease	0.002 (0.036)
Depression	0.067 (0.798)
Fear	0.081 (1.309)
Internal entrapment	0.242 (2.714)^**^
COVID history	0.511 (4.210)^**^
External entrapment	0.124 (1.454)

Dependent variable: Quitting jobs.

* *p* < 0.05;

** *p* < 0.01.

As per the results given in Table 9, COVID history significantly predicted quitting jobs (*β* = 0.511; *p <* 0.01). In addition to COVID History, Internal Entrapment (*β* = 0.242; *p <* 0.01) and Education (*β* = 0.147; *p <* 0.05) predicted quitting jobs. These variables also explained a significant proportion of variance in quitting jobs, *R*^2^ = 0.237, *F*(11, 224) = 7.648, *p <* 0.01.

## Discussion

The results about the fear of coronavirus which are given in Table 4 suggest that people do not consider themselves to be in the risk group or believe they would not die of coronavirus. They were simply afraid of causing the death of someone, be it a family member or not. The results also show that both internal and external entrapment feelings, and depression levels are correlated with gender. The literature supports statistically significant differences in depression levels and feelings of entrapment between genders. Previous studies also show that females have a higher entrapment level than men (O’Connor et al., 2021), and the occurrence and risk of depressive disorder are higher in females than in males (Piccinelli and Wilkinson, 2000; O’Connor et al., 2021).

The PEoC is correlated with COVID-19 history, and the mean difference of PEoC of moderate and highly affected groups is significantly higher than in the no-effect and mildly affected groups, which suggests that quitting jobs is higher among employees whose PEoC is moderate or high.

As far as the education level, both the correlation and regression analyses show that the education level affects quitting jobs. Although no research in the literature supports this finding, higher education levels may cause a greater perception of event strength, resulting in higher levels of emotional exhaustion (Liu et al., 2021) and, hence, quitting the job. Alternatively, it may be due to the employees’ financial status and savings. The level of income is closely related to the education level. The employees with a higher level of education may have savings upon which they can rely until the end of the pandemic, or it is taken under control, enabling them to consider quitting their jobs.

The correlation between the gender and PEoC of the infected employees is significant, which suggests that, when infected with COVID-19, the PEoC on women is worse than men, which needs to be verified in line with the values of other cultures. In the Turkish culture, working women also do housework at home, which may cause them to have a worse PEoC compared to men, even after recovery from the coronavirus. This difference, however, may not be attributed to genetics since women are favored in that respect. When compared with men, the genetic difference protects women against severe diseases (Bhatia et al., 2013), even in the case of coronavirus (Sharma et al., 2020). This may be an indication that the psychological recovery of women is taking longer time than that of men for, as shown in previous studies, females have a higher entrapment level than men (O’Connor et al., 2021), and the occurrence and risk of depressive disorder are higher in females than in males (Piccinelli and Wilkinson, 2000; O’Connor et al., 2021).

Although the previous studies report contradictory outcomes of working from home (Oakman et al., 2020), we found an interesting result. The correlation between depression scores and the work location of infected employees turned out to be statistically insignificant, which is significant for employees without coronavirus history. The employees with coronavirus history may have experienced the hardship of being infected and feeling safe when working from home, which is not the case with the employees without coronavirus history. Nevertheless, the depression level of the employees working only from home is significantly higher than the other groups. The literature supports this result since previous studies have shown that working from home causes employees to develop negative feelings and agoraphobia (Ahrentzen, 1989), and the problem *has no name* (Freiden, 1957).

Furthermore, perceiving working from the home situation as an ordinary home-office process may be misleading since all family members have been locked in during the COVID-19 period, and all activities, even education, are carried on online. No visitors, no visitings, no different activities, but work, and all family members tried to maintain work-life balance by using the same technological infrastructure. The fulfillment of work and home life responsibilities and socialization styles have changed or have been limited. These factors may have worsened the situation at home and increased the depression during the COVID-19.

For those who have COVID-19 history, the correlation was found to be significant between quitting jobs and the education level, internal and external entrapment, PEoC, fear, and depression. Interestingly enough, the Pearson correlation coefficients and significance levels of employees with coronavirus history are very low for fear and depression, suggesting that employees start to develop something like a “been there, done that” mood. This situation may also be related to the employees’ assurance that—now that they have recovered from COVID-19—their bodies have enough antibodies to fight against the viruses in case they ever contract it again.

Regression analysis results confirm that the COVID-19 history and internal entrapment effect the resignation. And also, the correlation was found to be significant between quitting jobs and the level of COVID-19 effect on life, internal entrapment, external entrapment, and depression. Although there are no previous studies to compare the results, this may be due to the hardship and psychological state that the coronavirus has caused (Piccinelli and Wilkinson, 2000; Elhai et al., 2020; Soheili et al., 2020; Zhang and Ma, 2020; Kumar, 2021; Liu et al., 2021; O’Connor et al., 2021; Parker and Clark, 2022, p. 34).

Although the correlation was found to be highest between quitting jobs and depression, the effect of depression on quitting the job could not be determined during regression analysis. The uncertainty, news, and rumors related to COVID-19 fuels anxiety (Elhai et al., 2020) and thus depression, which is mainly associated with anxiety.

### Theoretical Implications

The scarce research on quitting jobs during COVID-19, or The Great Resign, has given different causes. Some of them are burnout syndrome (BMA, 2021; Chugh, 2021; Sheather and Slattery, 2021), toxic organizational culture (Sull et al., 2022), the comfort of working from home (Chugh, 2021), insufficient salary or benefits (Hirsch, 2021; Parker and Clark, 2022), relocation (Birinci and Amburgey, 2022), reassessing priorities in life, and seeking for an elusive work-life balance (Kumar, 2021, p. 34). Employers and employees having a misaligned picture of factors driving job satisfaction and employee requirements are also counted among the reasons.

Not finding a significant correlation between work location and quitting the job makes us think that the comfort of working from home (Chugh, 2021) may not be the cause. Furthermore, considering the higher depression level of those working only from home eliminates that option as well. Most of the interviewed people did not have problems with the salary or benefits, but most of them had already quit or were seriously thinking of quitting. Therefore, the insufficient salary or benefits (Hirsch, 2021; Parker and Clark, 2022) should not be the cause as well.

This study, however, shows that there should be more to consider. Results show that fear of causing someone’s death, feeling entrapped, perceived effect of COVID-19 on worsened quality of life, and depression are highly associated with quitting the job.

### Recommendations for Further Research

Although there are several suggestions about the causes of the Great Resign, empirical studies need to be conducted to find the real cause(s). Especially qualitative studies will be able to shed light on the causes and missing variables, if there are any.

Moreover, reassessing life priorities, the changing perception of work and work-life balance, the desire to realize the dreams that are always postponed, and diminishing appetite for worldly materials may be considered or evaluated as causes of the great resign.

Along with entrapment and depression, the relation of alexithymia with quitting the job should also be investigated.

### Implications for Practice

One of the most critical aspects, which also needs to be carefully investigated and focused on, is increased depression. The mean depression level of the employees whose lives are highly affected by COVID-19 is close to moderate depression level, and this increase in the depression score suggests that psychological support should be provided to the employees whose life is being affected by the coronavirus. As per the results of this study, organizations willing to keep their key employees are urged to employ a psychologist or encourage their employees to visit psychologists to make them overcome the depression and the feeling of entrapment.

Considering the correlation between work location and depression, hybrid working conditions (working both from home and the workplace) or reducing the workload (including work hours) of those working only from home should be considered and assessed by the organizations.

Organizations should find a way to improve the PEoC of employees who have recovered from COVID-19 because the low perceived quality is associated with most mental health problems. Especially female employees, in this sense, require special consideration, for their internal and external entrapment and depression levels are higher than men.

Furthermore, because of the mental health problems their employees have gone through during the pandemic, organizations should be much more flexible with the employees and amend their policies and key performance indicators accordingly.

### Limitation

This study has its inherent limitations.

These limitations can be cited as follows: (1) it adopted an online, unadministered survey that could impact the given answers; (2) its sample size; and (3) it was conducted in one single location (Istanbul, Turkey), which may have limitations on generalizability. (4) It is also a cross-sectional study that adopted a convenient sampling method, which is another limitation. (5) It included only people who are being employed, which causes other limitations on generalizability. (6) This study was conducted during the pandemic.

Due to these limitations, the results should be interpreted carefully and accordingly.

## Conclusion

This study aims to investigate the factors associated with the resignation of employees during the COVID-19 period. The variables included in this study were selected based on the interviews conducted with the employees with coronavirus history.

This study showed that the correlation between quitting jobs and other conditions differs depending on the COVID-19 history of the employee. However, PEoC, depression, and internal and external entrapments are associated with quitting jobs for both infected and uninfected groups. Meanwhile, the PEoC was found to be worse for female employees with COVID-19 history and quitting jobs associated with higher education levels for the employees with COVID-19 history.

The regression analysis showed that the coronavirus history and the internal entrapment are the best predictors of the resignation. Furthermore, the education level also affects the resignation. Employees with PhD and MSc degrees tend to quit more than those with bachelor’s degrees and high-school diplomas.

## Data Availability Statement

The data that support the findings of this study are available from the corresponding author upon reasonable request.

## Ethics Statement

The studies involving human participants were reviewed and approved by Kocaeli University Social and Human Sciences Ethics Committee (protocol number: E-10017888-108.99-62960). The patients/participants provided their written informed consent to participate in this study.

## Author Contributions

HD, MA, and HG: conceptualization. MA, VD, and DR: methodology and validation. MA: formal analysis. HD, MA, HG, VD, and DR: data curation. HD and MA: writing—original draft preparation. HG, VD, and DR: writing—review and editing. HD: supervision and ethical commission permission. All authors contributed to the article and approved the submitted version.

## Conflict of Interest

The authors declare that the research was conducted in the absence of any commercial or financial relationships that could be construed as a potential conflict of interest.

## Publisher’s Note

All claims expressed in this article are solely those of the authors and do not necessarily represent those of their affiliated organizations, or those of the publisher, the editors and the reviewers. Any product that may be evaluated in this article, or claim that may be made by its manufacturer, is not guaranteed or endorsed by the publisher.

## References

[ref1] Ahrentzen S. (1989). A place of peace, prospect, and: the home as office. J. Architec. Plan. Res. 6, 271–288.

[ref2] Beck A. T. Steer R. A. Brown G. K. (1996). Beck Depression Inventory. 2nd Edn. San Antonio (TX): The Psychological Corporation.

[ref3] Bhatia K. Zimmerman M. A. Sullivan J. C. (2013). Sex differences in angiotensin-converting enzyme modulation of Ang (1-7) levels in normotensive WKY rats. Am. J. Hypertens. 26, 591–598. doi: 10.1093/ajh/hps088, PMID: 23547034 PMC3657482

[ref4] Birinci S. Amburgey A. (2022). The great resignation vs. The Great Reallocation: Industry-Level Evidence. Eco. Synopses 4, 1–3. doi: 10.20955/es.2022.4

[ref5] Bloomberg Businessweek (2021). From the great resignation to lying flat, workers are opting out. Available at: https://www.bloomberg.com/news/features/2021-12-07/why-people-are-quitting-jobs-and-protesting-work-life-from-the-u-s-to-china (Accessed February 12, 2022).

[ref6] BMA (2021). Thousands of Overworked Doctors plan to leave the NHS, BMA finds. Available at: https://www.bma.org.uk/bma-media-centre/thousands-of-overworked-doctors-plan-to-leave-the-nhs-bma-finds (Accessed February 12, 2022).

[ref7] Boratyńska K. (2021). A new approach for risk of corporate bankruptcy assessment during the COVID-19 pandemic. J. Risk Financ. Manage. 14:590. doi: 10.3390/jrfm14120590

[ref8] Chaturvedi P. Chaturvedi A. Singh A. G. (2021). I COVID-19 pandemic: A story of helpless humans, confused clinicians, rudderless researchers, and a victorious virus! Cancer Res. Statistics Treatment 4:1. doi: 10.4103/crst.crst_367_20

[ref9] Chugh A. (2021). What is ‘The Great Resignation’? An expert explains. World Economic Forum. Available at: https://www.weforum.org/agenda/2021/11/what-is-the-great-resignation-and-what-can-we-learn-from-it (Accessed February 12, 2022).

[ref10] Da-Silva-Lopes B. C. Gil-da-Silva-Lopes P. Jaspal R. (2021). Exposure to COVID-19 risk representations and state depressive symptoms in a United Kingdom sample: A preliminary experimental study (Representaciones de riesgos referentes a la exposición al COVID-19 y síntomas depresivos actuales en una muestra del Reino Unido: Un estudio experimental preliminar). Stud. Psychol. 42, 615–651. doi: 10.1080/02109395.2021.1950461

[ref11] Davidson P. (2021). Great Resignation: The Number of People Quitting Jobs hit an all-time high in November as Openings Stayed near Record. USA Today. January 4.

[ref12] Elhai J. D. Yang H. McKay D. Asmundson G. J. G. (2020). COVID-19 anxiety symptoms associated with problematic smartphone use severity in Chinese adults. J. Affect. Disord. 274, 576–582. doi: 10.1016/j.jad.2020.05.080, PMID: 32663990 PMC7251360

[ref13] Flett G. L. Hewitt P. L. (2020). The perfectionism pandemic meets COVID-19: understanding the stress, distress, and problems in living for perfectionists During the Global Health crisis. J. Concurrent Dis. 2, 80–105. doi: 10.54127/AXGJ8297

[ref14] Freiden B. (1957). The Feminine Mystique. New York: W.W. Norton.

[ref15] Gilbert P. Allan S. (1998). The role of defeat and entrapment (arrested flight) in depression: An exploration of an evolutionary view. Psychol. Med. 28, 585–598. doi: 10.1017/S0033291798006710, PMID: 9626715

[ref16] Hirsch P. B. (2021). The great discontent. J. Bus. Strateg. 42, 439–442. doi: 10.1108/JBS-08-2021-0141, PMID: 34758135

[ref17] Kumar G. B. (2021). The Great Resignation: American Workers Suffering a Crisis of Meaning. United States: The Rand Corporation.

[ref18] Lee H.-J. Park B.-M. (2021). Feelings of entrapment during the COVID-19 pandemic based on ACE star model: A concept analysis. Healthcare 9:1305. doi: 10.3390/healthcare9101305, PMID: 34682983 PMC8544561

[ref19] Liu Y. Zhang Z. Zhao H. (2021). The influence of the COVID-19 event on deviant workplace behavior taking Tianjin, Beijing and Hebei as an example. Int. J. Environ. Res. Public Health 18:59. doi: 10.3390/ijerph18010059, PMID: 33374789 PMC7794894

[ref21] O’Connor R. C. Wetherall K. Cleare S. McClelland H. Melson A. J. Niedzwiedz C. L.. (2021). Mental health and well-being during the COVID-19 pandemic: longitudinal analyses of adults in the UK COVID-19 Mental Health & Wellbeing study. Br. J. Psychiatry 218, 326–333. doi: 10.1192/bjp.2020.212, PMID: 33081860 PMC7684009

[ref22] Oakman J. Kinsman N. Stuckey R. Graham M. Weale V. (2020). A rapid review of mental and physical health effects of working at home: how do we optimise health? BMC Public Health 20:1825. doi: 10.1186/s12889-020-09875-z, PMID: 33256652 PMC7703513

[ref23] Parker R. Clark B. Y. (2022). Unraveling the great resignation: impacts of the COVID-19 pandemic on Oregon workers. Available at: https://papers.ssrn.com/sol3/papers.cfm?abstract_id=4019586 (Accessed January 27, 2022).

[ref24] Piccinelli M. Wilkinson G. (2000). Gender differences in depression: critical review. Br. J. Psychiatry 177, 486–492. doi: 10.1192/bjp.177.6.486, PMID: 11102321

[ref25] Sharma G. Volgman A. S. Michos E. D. (2020). Sex differences in mortality from COVID-19 pandemic: are men vulnerable and women protected? JACC: Case Rep. 2, 1407–1410. doi: 10.1016/j.jaccas.2020.04.027, PMID: 32373791 PMC7198137

[ref26] Sheather J. Slattery D. (2021). The great resignation—how do we support and retain staff already stretched to their limit? BMJ 375:n2533. doi: 10.1136/bmj.n2798, PMID: 34663568

[ref27] Siddaway A. P. Taylor P. J. Wood A. M. Schulz J. (2015). A meta-analysis of perceptions of defeat and entrapment in depression, anxiety problems, post-traumatic stress disorder, and suicidality. J. Affect. Disord. 184, 149–159. doi: 10.1016/j.jad.2015.05.046, PMID: 26093034

[ref28] Smarr K. L. Keefer A. L. (2011). Measures of depression and depressive symptoms: Beck depression inventory-II (BDI-II), Center for Epidemiologic Studies Depression Scale (CES-D), geriatric depression scale (GDS), hospital anxiety and depression scale (HADS), and patient health Questionnaire-9 (PHQ-9). Arthritis Care Res. 63, S454–S466. doi: 10.1002/acr.20556 22588766

[ref29] Soheili S. Shariat A. Anastasio A. T. (2020). Modification of existing occupational therapeutic protocols in response to the “new Normal” after COVID-19: letter to the editor. Work 66, 477–478. doi: 10.3233/WOR-203192, PMID: 32651341

[ref30] Sull D. Sull C. Zweig B. (2022). Toxic culture is driving the great resignation. MIT Sloan Manag. Rev. 63, 1–9.

[ref31] Taylor P. J. Gooding P. Wood A. M. Tarrier N. (2011). The role of defeat and entrapment in depression, anxiety, and suicide. Psychol. Bull. 137, 391–420. doi: 10.1037/a0022935, PMID: 21443319

[ref32] Üngüren E. Ceyhan S. Türker N. (2022). How does fear of COVID-19 affect the mental well-being of waiters in Turkey. Work, 1–12. [Epub Ahead of Print]. 10.3233/WOR-211030 35253713

[ref33] WHO (2022). Coronavirus disease (COVID-19) pandemic. Available at: https://www.who.int/emergencies/diseases/novel-coronavirus-2019 (Accessed February 12, 2022).

[ref34] Zhang Y. Ma Z. F. (2020). Impact of the COVID-19 pandemic on mental health and effect of COVID-19 among local residents in Liaoning Province, China: A cross-sectional study. Int. J. Environ. Res. Public Health 17:2381. doi: 10.3390/ijerph17072381, PMID: 32244498 PMC7177660

